# A genetic variant of the NTCP gene is associated with HBV infection status in a Chinese population

**DOI:** 10.1186/s12885-016-2257-6

**Published:** 2016-03-12

**Authors:** Jingmin Yang, Yuan Yang, Mingying Xia, Lianghui Wang, Weiping Zhou, Yajun Yang, Yueming Jiang, Hongyang Wang, Ji Qian, Li Jin, Xiaofeng Wang

**Affiliations:** Epidemiology unit of MOE Key Laboratory of Contemporary Anthropology and State Key Laboratory of Genetic Engineering, School of Life Sciences and Institutes of Biomedical Sciences, Fudan University, 220 Handan Rd., Shanghai, 200433 China; China Medical City Institute of Health Sciences, 1 Yaocheng Road, Taizhou, Jiangsu 225300 China; Eastern Hepatobiliary Surgery Hospital, Second Military Medical University, Shanghai, 200438 China; Department of Health Statistics, Second Military Medical University, Shanghai, 200433 China; National Innovation Alliance for Hepatitis & Liver Cancer, Shanghai, 200438 China; Cancer Institute, Fudan University Shanghai Cancer Center, Shanghai, 200032 China; Department of Oncology, Fudan University Shanghai Medical College, Shanghai, 200032 China

**Keywords:** HBV-receptor, NTCP, Genetic variant, HBV infection status, Association, rs4646287

## Abstract

**Background:**

To investigate whether genetic variants of the HBV receptor gene NTCP are associated with HBV infection in the Han Chinese population.

**Methods:**

We sequenced the entire 23 kb NTCP gene from 111 HBeAg-positive HBsAg carriers (PSE group), 110 HBeAg-negative HBsAg carriers (PS group), and 110 control subjects. Then, we performed association analyses of suggestively significant SNPs with HBV infection in 1075 controls, 1936 PSs and 639 PSEs.

**Results:**

In total, 109 rare variants (74 novel) and 38 single nucleotide polymorphisms (SNPs, one novel) were screened. Of the seven non-synonymous rare variants, six were singletons and one was a double hit. All three damaging rare singletons presented exclusively in the PSE group. Of the five SNPs validated in all 3650 subjects, the T allele of rs4646287 was significantly decreased (*p* = 0.002) in the PS group (10.1 %) and PSE group (8.1 %) compared to the controls (10.9 %) and was decreased to 7.4 % in the PSE hepatocellular carcinoma (HCC) subgroup. Additionally, rs4646287-T was associated with a 0.68-fold (95 % *CI* = 0.51–0.89, *p* = 0.006) decreased risk of PSE compared with the controls. The NTCP mRNA level was lower in HCC tissues in “CT + TT” carriers than in “CC” carriers.

**Conclusions:**

We found a genetic variant (rs4646287) located in intron 1 of NTCP that may be associated with increased risk of HBV infection in Han Chinese.

**Electronic supplementary material:**

The online version of this article (doi:10.1186/s12885-016-2257-6) contains supplementary material, which is available to authorized users.

## Background

Hepatitis B virus (HBV) infection is a major global public health issue. Serological evidence of HBV infection was observed in approximately one-third of the world’s population, of which 350–400 million people were chronically infected [1]. Individuals infected with HBV are at high risk for the development of cirrhosis, liver failure, or hepatocellular carcinoma (HCC) [2, 3]. HBV is a DNA virus that primarily infected hepatocytes which mediated by HBV receptors located on the hepatocyte cell membrane. Although identifying the HBV receptors is not easy, several candidates have been reported [4–6].

Recently, Li et al. found that NTCP (sodium taurocholate cotransporting polypeptide), which was specifically expressed in hepatocytes, was a necessary cell surface receptor for HBV entry [7]. NTCP specifically interacts with the receptor-binding region of HBV pre-S1 and mediates woolly monkey HBV infection of *Tupaia* hepatocytes [7, 8]. Silencing NTCP inhibited HBV and HDV infection, whereas exogenous NTCP expression rendered non-susceptible hepatocarcinoma cells susceptible to viral infection [7]. Additionally, Dieter Glebeb et al. found that tent-making bat HBV (TBHBV) could infect human hepatocytes using the human NTCP (hNTCP) receptor [9].

Host genetics play an important role in the clinical outcome of HBV infection. Due to the popularity of genome-wide association studies (GWAS) in recent years, several susceptibility loci associated with HBV-related diseases have been identified. For example, genetic variants in the HLA-DP locus were found to be strongly associated with a risk of persistent HBV infection [10]. Several genetic susceptible loci for HBV-related HCC were also identified [11–13]. However, the relationship between genetic variants of full-length *NTCP* and the HBV infection status has rarely been investigated [14, 15]. In this study, we investigated whether genetic variants in the *NTCP* gene predisposed subjects to HBsAg + HBeAg-positivity (representing higher HBV activity) and HBsAg-positivity (representing lower HBV activity) based on the finding that a higher serum HBV DNA concentration was observed in HBeAg-positive compared to HBeAg-negative individuals [1, 16, 17].

## Methods

### Subjects

A population-based study wad designed with three groups for comparison, including 639 subjects positive for HBsAg and HBeAg (the PSE group), 1936 subjects positive for HBsAg (the PS group) and 1075 subjects negative for HBV antibodies and antigens (the control group). All of the study subjects were East China residents. A total of 2647 subjects were randomly selected from the hepatitis sub-cohort (including 43,000 subjects) of the Taizhou longitudinal (TZL) study. The profile of the TZL study was described elsewhere [18]. An additional 299 subjects with hepatocellular carcinoma for the PSE group and 704 subjects with hepatocellular carcinoma for the PS group (HCC, long-term outcome of persistent infection of HBV) were recruited from the Shanghai Eastern Hepatobiliary Surgery Hospital (EHBH). The ages of the subjects in the PSE group, PS group, and control group were 45.34 ± 11.55 yrs, 52.36 ± 11.35 yrs, and 53.53 ± 12.93 yrs, respectively, and the three groups included 67.3, 65.6, and 49.5 % males, respectively. For the functional analysis, 33 paired tumor/non-neoplastic adjacent (normal) liver tissues from HBV-related HCC patients were obtained from the EHBH. Written informed consent was acquired from all of the participants, and the study protocol was approved by the Ethics Committee of the School of Life Sciences of Fudan University.

### Identification of SNPs/indels in NTCP by Sanger sequencing

#### DNA preparation

Genomic DNA was isolated from human peripheral blood lymphocytes using the Mini DNA Extraction kit for Peripheral Blood (Lifefeng, Shanghai, China) and quantified with the NanoDrop ND-1000 spectrophotometer (NanoDrop, Wilmington, DE, USA).

#### Primer design

A full-length of 23,872 bp sequence of the human *NTCP* gene was obtained from the GENE database of NCBI (National Center for Biotechnology Information, RefSeq: NC_000014.8) for primer design. Overall, 42 pairs of PCR primers were designed to amplify this gene, including the full-length gene and the core promoter, located from 70,240,928 to 70,264,606 on chromosome 14. The lengths of the PCR amplicons ranged from 725 bp to 1821 bp. The forward primers and/or reverse primers for each amplicon were used as Sanger sequencing primers. Furthermore, several nested primers were also used as the sequencing primers for the two long amplicons [Additional file 1].

#### PCR-based Sanger sequencing of NTCP

The PCR products of each primer pair were sequenced using an ABI 3730xl DNA Analyzer (Applied Biosystems, Foster City, CA, USA). Overall, 111, 110 and 110 subjects were chosen randomly from the PSE, PS, and control groups, respectively, for variant sequencing and variant frequency comparisons among groups.

#### SNP and indel identification

SNPs were identified utilizing the software CodonCode Aligner (CodonCode Corporation, Centerville, MA, USA), and indels were deduced manually after aligning to the reference sequence.

### SNP genotyping assay

The variants for genotyping were chosen according to the following criteria: (1) SNPs distributed significantly (p < 0.05) or marginally significantly (p < 0.06) between any two groups (control, PS, PSE, and PS + PSE groups); (2) non-synonymous SNPs; (3) selected SNPs in the main LD block; and (4) novel variants that occurred only in the PSE + PS or controls at least three times.

The TaqMan SNP genotyping assay was performed to genotype the chosen variants in 528 PSE subjects, 1826 PS subjects and 965 Controls. All samples were genotyped using TaqMan on an ABI GeneAmp® PCR system 9700 (Applied Biosystems, Foster City, CA, USA) and an ABI Prism 7900HT sequence detection system (Applied Biosystems, Foster City, CA, USA).

### Analysis of gene expression using real-time PCR

Total RNA was extracted from paired tumour and normal liver tissues from HBV-related HCC patients with the TRIzol reagent (Invitrogen, Carlsbad, CA, USA) [19] according to the manufacturer’s protocol. The concentration of the isolated RNA and the A260/A280 ratio were measured with the NanoDrop ND-1000 spectrophotometer (NanoDrop Technologies, Wilmington, DE, USA). Then, 1 μg of the total RNA was used to synthesize first-strand cDNA with the PrimeScript™ RT reagent Kit (TaKaRa Bio, JPN). These products were diluted 1:40, and 2 μl was used as the template in a 10 μl real-time PCR reaction performed on an ABI Prism 7900 HT Sequence Detection System (Applied Biosystems, Foster City, CA, USA) using the SYBR® Premix Ex Taq™ II (TaKaRa Bio, JPN). The housekeeping gene *ACTB* was adopted as the reference gene for the real-time PCR. Primers spanning the introns were designed using the Primer Express™ software (Applied Biosystems, Foster, CA, USA). The primer sequences were as follows:

ACTB: Forward (5′-GCTCCGGCATGTGCAA-3′);

Reverse (5′TCGCCCACATAGGAATCCTT-3′)

NTCP: Forward (5′-GGACATGAACCTCAGCATTGTG-3′);

Reverse (5′-ATCATAGATCCCCCTGGAGTAGAT-3′).

### Statistical analysis

Deviation from the Hardy–Weinberg equilibrium (HWE) expectation for any genetic variant was tested using a Chi-square statistic. Continuous variables were expressed as the means (± standard deviation), and comparisons of continuous variables were conducted using the Student’s *t*-test. Unconditional logistic regression analyses were used to estimate the odds ratios (ORs) and their 95 % confidence intervals (CIs) for PS (HBsAg-positivity) or PSE (HBsAg- and HBeAg-positivity) under the assumption of different genetic models. Multivariate analyses were performed to adjust for age and gender. SPSS software version 16.0 for Windows was used in the statistical evaluation of the above-mentioned data.

The *NTCP* mRNA expression levels were calculated using the 2^-Δct^ method. The *NTCP* mRNA expression levels were calculated relative to the ACTB housekeeping gene. The fold changes in the *NTCP* mRNA levels of tumor tissues relative to their paired normal tissues were calculated using the 2^-ΔΔct^ method.

To construct relevant haplotypes, the genotype data were used to estimate the inter-marker LD, measure the pair-wise D’ and r^2^ and define the LD blocks. Haplotype inferences and haplotype association tests were estimated using Haploview [20].

Because the sample size was relatively small, a suggestive significance threshold of P < 0.05 was used to select SNPs for variant discovery. The Bonferroni correction was used to weed out false positive findings due to multiple testing. A significance threshold of P < 0.01 was used after multiplication by the total number of genotyped SNPs (0.05/5).

## Results

### Variants discovered by sequencing

We sequenced ~23 kb of the *NTCP* gene, including all five exons, four introns, as well as the core promoter region [21], and a 1.2 kb region downstream of the *NTCP* gene with high success rates. The exception was six regions (~3 kb) located in the first and second introns around the highly repetitive elements (i.e., Alu repeats and homo-polymers). Overall, 147 variants, including 109 rare variants (74 novel) and 38 SNPs (one novel), were detected [see Additional file 2]. Approximately 91.8 % (135) of the variants were located in non-exon regions, with 38.8 % (30) in the first intron, 33.3 % (22) in the second intron and 11.6 % (17) in downstream (16) and upstream (1) regions. Moreover, the MAF (minor allele frequency) of 74.1 % (109) and 15.0 % (22) of the variants lied below 1 % and ranged from 10 to 20 %, respectively [see Additional file 3].

Among the 10 variants in the coding sequence, eight were non-synonymous, and two were synonymous variants; six of the non-synonymous and one of the synonymous variants were singletons. The effects of non-synonymous coding variants on protein functions were predicted by SIFT [22] and PolyPhen [23] (http://www.snp-nexus.org/) (Table 1). The variants 333A > T and 88I > T (both of which were predicted to be tolerated/benign by SIFT/Polyphen) were not found in the PSE group. Correspondingly, three variants (256 M > T, 222 L > S, and 200 V > M) predicted to be damaging/possibly damaging presented exclusively in the PSE group. Furthermore, a rare SNP 267S > F variant (rs2296651) that was predicted to be probably damaging by Polyphen and to cause a near complete loss of function for bile acid uptake of NTCP [24] occurred more frequently in the PSE group. Based on these observations, more damaging mutations were found in PSE group than the control or PS groups.

**Table 1:** Non- synonymous variants identified in the sequencing results

Variants	AA change	Novel	Exon	Sift Prediction		Polyphen Prediction		MAF (%)		
				Score	Prediction	Score	Prediction	Control	PS	PSE
+21059 C/T	333A > T	Yes	5	0.28	Tolerated	0	Benign	0	0.5	0
+21029 C/G	323A > P	Yes	5	0.08	Tolerated	0.476	Possibly damaging	0.5	0	0
rs2296651	267S > F	No	4	0.31	Tolerated	0.88	Probably damaging	0.9	0.9	2.3
+18885 A/G	256 M > T	Yes	4	0	Damaging	0.839	Possibly damaging	0	0	0.5
+18131 A/G	222 L > S	Yes	3	0	Damaging	0.637	Possibly damaging	0	0	0.5
rs202213974	200 V > M	No	3	0	Damaging	0.819	Possibly damaging	0	0	0.5
+11121 G/C	131 L > V	Yes	2	0.23	Tolerated	0.484	Possibly damaging	0.5	0	0
rs148467625	88I > T	No	1	0.57	Tolerated	0.072	Benign	0.5	0.5	0

### SNP rs4646287 and HBV infection

Based on the validation criteria described in the Materials and Methods, six SNPs from the identified variants were chosen for genotyping in all subjects. Among the six SNPs, SNP rs2296651 was a non-synonymous damaging variant. SNP rs4646287 exhibited difference between the PSE and control groups with marginal statistical significance. Nineteen common SNPs were used to construct a large haplotype block (r^2^ ranged from 0.74 to 1) spanning approximately 21 kb; three of these SNPs (rs4646285, rs28437822 and rs11624523) were chosen, of which rs4646285 was located in exon 1. The rare variant +20988G > A occurred three times only in the PSE + PS group.

**Fig. 1:**
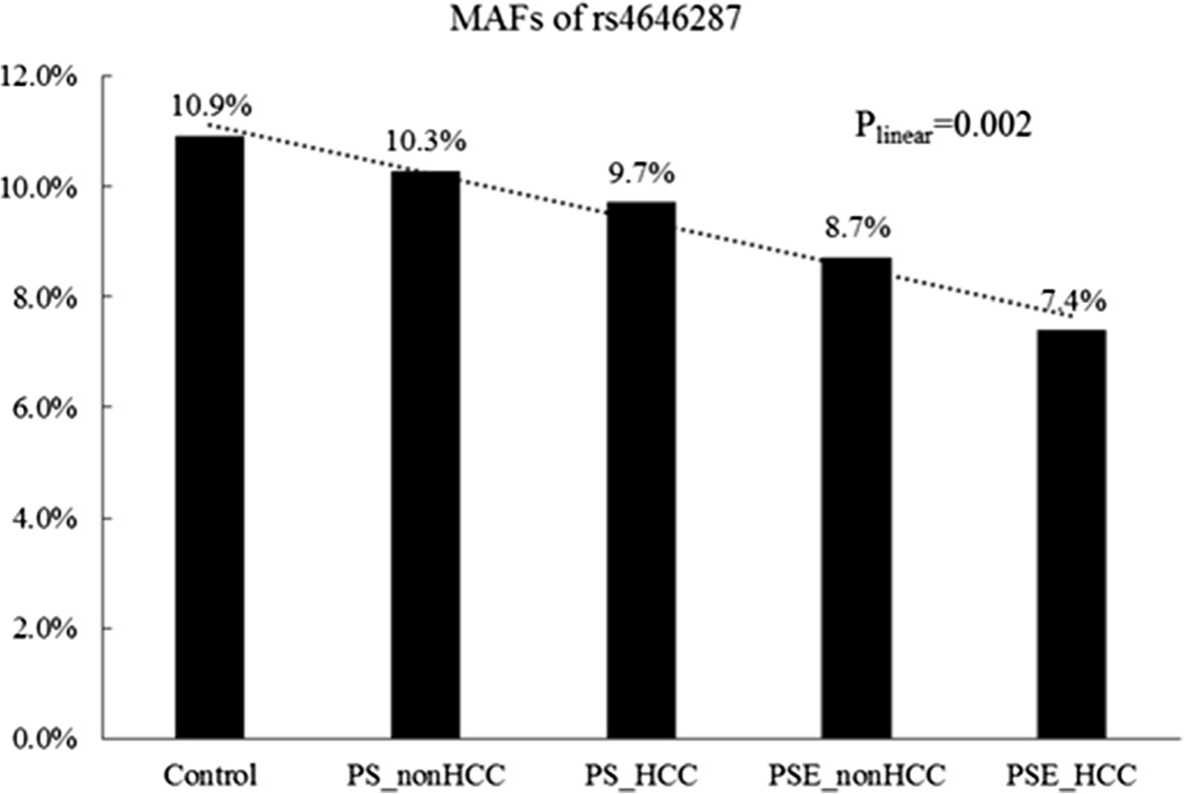
MAFs of rs4646287 in different groups or subgroups

Because the homozygote mutant TT genotype frequency of rs4646287 was only 0.7, 1.3, and 1.2 % in the control, PS, and PSE groups, respectively, we combined the rare homozygotes and heterozygotes for further analysis in a dominant model. The T allele was associated with a decreased risk of PSE with a MAF of 8.1 % compared with the control group that had a MAF of 10.9 % (*OR* = 0.67, 95 % CI: 0.51–0.87, *p* = 0.003, Table 2). The association remained after adjusting for age and gender (*OR* = 0.68, 95 % CI (0.51–0.79), *p* = 0.006). Additionally, the T allele frequencies decreased significantly from the Control (10.9 %), to the HCC (−) PS (10.3 %) subgroup, HCC (+) PS subgroup (9.7 %), HCC (−) PSE (8.7 %) subgroup and HCC (+) PSE subgroup (7.4 %) (p_linear_ =0.002) (Fig. 1).

**Table 2:** SNPs associated with PSE and their odds ratios relative to the control

SNP	Control, *N* = 1075(100 %)	PS, *N* = 1936 (100 %)	PSE, 639 (100 %)	P_linear_	PSE vs Control		
					*P*	OR	95 % CI
rs4646287							
CC	843 (79.0)	1548 (81.2)	541 (84.9)		0.005	1	
CT	215 (20.1)	334 (17.5)	89 (14.0)		0.0014	0.65	0.49–0.85
TT	9 (0.8)	25 (1.3)	7 (1.1)		0.705	1.21	0.45–3.27
CT + TT	224 (21.0)	359 (18.8)	96 (15.1)	0.004	0.0026	0.67	0.51–0.87
C	1901 (89.1)	3430 (89.9)	1171 (91.9)			1	
T	233 (10.9)	384 (10.1)	103 (8.1)	0.010	0.0075	0.72	0.56–0.92
rs2296651							
GG	1010 (94.7)	1834 (95.2)	585 (91.7)			1	
GA	57 (5.3)	92 (4.8)	53 (8.3)	0.033	0.017	1.61	1.09–2.37
G	2077 (97.3)	3760 (97.6)	1223 (95.8)			1	
A	57 (2.7)	92 (2.4)	53 (4.2)	0.036	0.021	1.58	1.05–2.35
rs28437822							
AA	769 (72.1)	1383 (72.2)	470 (73.7)		0.062	1	
AG	266 (25.0)	491 (25.6)	161 (25.2)		0.933	0.99	0.79–1.24
GG	31 (2.9)	41 (2.1)	7 (1.1)		0.018	0.37	0.16–0.85
AG + GG	297 (27.9)	532 (27.7)	168 (26.3)	0.542	0.493	0.93	0.74–1.16
A	1804 (84.6)	3257 (85.0)	1101 (86.3)			1	
G	328 (15.4)	573 (15.0)	175 (13.7)	0.208	0.195	0.87	0.71–1.07
rs11624523							
AA	766 (71.5)	1380 (72.0)	467 (73.7)		0.153	1	
AG	272 (25.4)	494 (25.8)	157 (24.8)		0.638	0.95	0.75–1.19
GG	33 (3.1)	43 (2.2)	10 (1.6)		0.056	0.50	0.24–1.02
AG + GG	305 (28.5)	537 (28.0)	167 (26.3)	0.372	0.341	0.90	0.72–1.12
A	1804 (84.2)	3254 (84.9)	1091 (86.0)			1	
G	338 (15.8)	580 (15.1)	177 (14.0)	0.110	0.166	0.87	0.71–1.06
rs4646285							
CC	768 (72.2)	1391 (72.5)	469 (73.7)		0.051	1	
CT	264 (24.8)	486 (25.3)	160 (25.2)		0.948	0.99	0.79–1.25
TT	32 (3.0)	42 (2.2)	7 (1.1)		0.015	0.36	0.16–0.82
CT + TT	296 (27.8)	528 (27.5)	174 (27.3)	0.736	0.484	0.92	0.74–1.15
C	1800 (84.6)	3268 (85.1)	1098 (86.3)			1	
T	328 (15.4)	570 (14.9)	174 (13.7)	0.182	0.178	0.87	0.71–1.06

### NTCP expression differences among rs4646287 genotypes

**Fig. 2:**
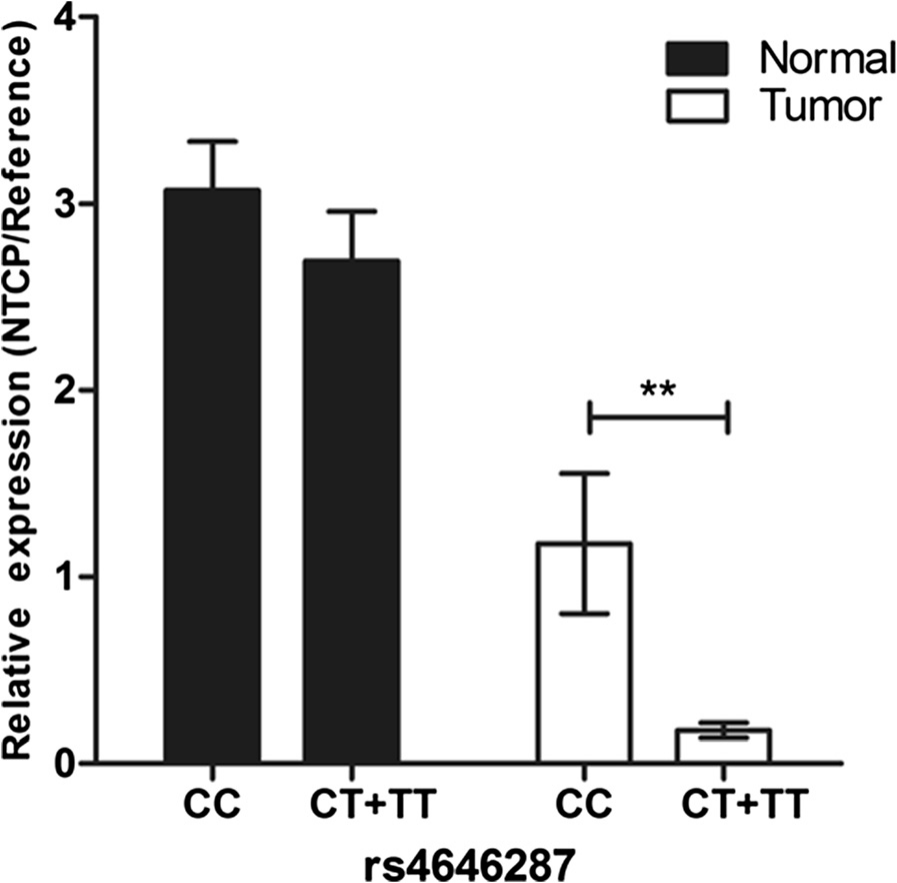
*NTCP* mRNA levels of different genotypes of rs4646287 in paired tumor/normal liver tissues. The values were normalized using the average level of ACTB, and the means (95 % CI) were shown. **: *p* value < 0.01

To evaluate the functional effects of rs4646287 on the transcriptional activity of NTCP, 33 paired tumor/normal liver tissues from HCC patients (16 CC, 15 CT, and 2 TT genotypes) were recruited to measure the *NTCP* mRNA levels using the real-time fluorescence quantitative PCR method. The clinical information for the samples was provided in Additional file 4. NTCP mRNA levels were lower in subjects with CT + TT genotypes than those with the CC genotype, although the difference was nearly 4.8 fold in tumor tissues but only 10 % in normal tissues (Fig. 2, Additional file 5).

### Other SNPs and HBV infection

We validated three SNPs (rs4646285, rs11624523, and rs28437822) among the 19 SNPs used to construct the large LD block. However, non of these SNPs exhibited a significant difference among the PSE, PS, and control groups. The non-synonymous SNP rs2296651 resulted in an amino acid change from serine to phenylalanine at position 267 (267S > F) of the NTCP protein. The frequencies of the A allele were 2.67, 2.57, and 4.15 % in the control, PS, and PSE groups, respectively. However, we did not find any significant differences in the A allele frequency among the three groups after adjusting for multiple testing.

### The rare variant +20988G > A occurred only in the PSE + PS group

The variant +20988G > A located in intron 4, 18 bp upstream of exon 5 arose only in the PSE + PS subjects. This variant occurred twice in the PS group and once in the PSE group among the sequenced subjects and five times in the PS group and twice in the PSE group in the genotyped subjects. In contrast, it was not observed in the controls.

## Discussion

In the present work, we described a genetic profile of the *NTCP* gene in a normal population, a HBe-negative population, and a HBe-positive population of Han Chinese. The rare and common genetic variants identified in the *NTCP* gene call for additional functional and trait association analyses. A functional genetic variant of *NTCP* (rs4646287) that was found to be associated with HBV infection status. The rare T allele of the SNP rs4646287 was not only linearly decreased in frequency in the control, PS, PSE and HCC subgroups but also predisposed to a decreased risk for PSE, which was indicative of reduced HBV activity. This result suggests a protective effect for the rs4646287-T allele for the host in response to HBV infection.

For the persistently infected patients in our study, the frequency of rs4646287 TT (or AA in the forward strand) was observed to be reduced (although not to a significant level) in the HCC group compared to the non-HCC group [10 (1.0 %) vs. 22 (1.4 %), *p* = 0.47 under a recessive model]. Consistent with this finding, the frequency of the TT genotype was 0.7 % in the HCC group and 1.4 % in the non-HCC group in a GWAS study (data not published). However, the frequency of the rs4646287 TT genotype was slightly higher in the HCC group than in the non-HCC group in a study that included 186 HCC and 395 non-HCC subjects [6 (3.2 %) vs. 1 (0.3 %) subjects, *p* = 0.018 in a recessive model][14]. This discrepancy could be explained by the relatively small sample size or the different construction of the aforementioned study. Indeed, the TT genotype was observed to be more common in the HCC group than in the non-HCC group [4 (1.3 %) vs. 3 (0.9 %)] in the HBeAg-positive subjects (PSE group) in our study, although the difference did not reach statistical significance.

The SNP rs4646287, which is located in the first intron approximately 1 kb downstream of the first exon, may be an important variant that influences *NTCP* expression. Genetic variants in the first intron may be important for the predisposition of complex traits. These variants may be located in an intron-based transcription enhancer or silencer, with different alleles corresponding to different gene expression levels [25–27]. Using the transcriptional factor binding site prediction software TFSEARCH [28], we found that C > T introduced a new DNA binding site for p300 (5′-AGGAG**C**G-3′ to 5′-AGGAG**T**G-3′), which might influence the expression of *NTCP* and suggested a functional significance for this variant. However, “CC” carriers exhibited increased *NTCP* mRNA levels compared to “CT + TT” carriers in normal tissues, and the lack of significance observed for this difference might be limited by the small sample size of our functional analysis (*n* = 33). Interestingly, the decreased *NTCP* mRNA levels of “CT + TT” compared to “CC” carriers reached as low as 0.21-fold in the tumor tissues, which may be due to the complicated enlargement effects of the pathological status.

The potential functional variant rs4646287 that influenced the HBV infection status might have evolutionary implications. The frequencies of the rs4646287 T allele differed among different ethnic groups; the frequency was 0 % in Africans and Europeans, but the allele was common in East Asians (11.31 % in Chinese and 12.35 % in Japanese populations [frequency information was obtained from the HapMap population data] and 10.93 % in our control Han subjects). However, the frequencies of the T allele in the HBV-infected subjects decreased to 10.10 % (PS group) and further decreased to 8.08 % in subjects with a more severe status (PSE group). Because Asians are more likely to be infected with HBV, the frequency of the functional rs4646287-T was decreased in the control, PS, and PSE subjects. Because rs4646287-T decreases the expression and hence function of NTCP, these results suggest that rs4646287-T is a protective factor in response to the selective pressure of HBV infection in Asians that arose during the long process of human evolution.

Notably, although the increased presence of the rs2296651 (Ser267Phe) mutant allele in the PSE group compared to the PS or control group did not survive multiple testing correction, this SNP was worth particular consideration. Phe267 is differentially distributed in different ethnic groups, with 0 % detected in Africans and Caucasians and a frequency of approximately 3–4 % in East Asians. Interestingly, the frequency of Phe267 decreases from southern to northern Chinese population, with 8.3 % in Guangdong and 8.1 % in Guangxi province (Southern China), 7.5 % in Chongqing, 5.4 % in Hunan and Hubei provinces, and 2.4 % in Henan province in a cohort of hepatitis B patients undergoing therapy with interferon a2a (data not shown). Functional analysis revealed that the mutant allele had a deleterious effect on the transport of bile acids [24]. Moreover, this mutation also reduced the chance of HBV entering hepatocytes [29]. This observation indicated that the mutant allele might be lower in HBV carriers, which was confirmed by the study of Yiming Wang et. al. who found that the Ser267Phe variant was significantly associated with resistance to CHB [15]. In the present study, we found that the frequency of the mutant A allele was decreased in the PS group and increased in the PSE group relative to the controls. This finding was interesting, although we did not find an association between the Ser267Phe variant and the lower risk of CHB, possibly due to the lower frequency of Ser267Phe in our population (Eastern China, MAF_control_ = 2.7 %) compared to Yiming Wang’s population (Southern China, MAF_control_ = 10.9 %) [15]. The frequency of HBV genotype C was reported to be higher in the PSE group than in the PS group in Han Chinese [30, 31]. Therefore, questions remain concerning whether the more invasive HBV-C prefers human rs2296651-A, resulting in a higher A in the PSE group. Our data showed that individuals with the rs2296651 GA genotype had a higher risk of infection with HBV-C than individuals with the GG genotype (*OR* = 1.42 *p* = 0.75), although this difference did not reach statistical significance. Thus, this mutant allele might affect the interaction between HBV and its receptor NTCP.

Rare variants (especially damaging variants predicted by SIFT/PolyPhen) are more frequently presented in the PSE group. Three of the eight missense variants predicted to be damaging, including Met256Thr (+18885 A/G, novel) in the fourth exon and Leu222Ser (+18131 A/G, novel) and Val200Met (rs202213974) in the third exon, were observed only in the PSE group. Neighbouring variants may induce several missense mutations that significantly reduce the uptake of bile acid and/or estrone sulfate by NTCP [24]. These mutations include Ile223Thr (rs61745930) in the third exon and Ser267Phe (rs2296651), Ile279Thr (rs72547507) and Lys314Glu (rs72547506) in the fourth exon. The above observations may imply that the third or fourth exon of *NTCP* plays an important role in NTCP substrate transport and that missense mutations in these regions can easily have deleterious impacts on the transport function of NTCP. Similarly, the Leu222Ser, Val200Met, and Met256Thr variants discovered in the present study may lead to reduced bile acid transport activity. Coincidently, rare variants identified in other exons (131 L > V in exon 2, 88I > T in exon 1, and 323A > P and 333A > T in exon 5) predicted to be benign/tolerated were not accumulated in the third or fourth exons. Larger studies are needed to evaluate the pathological effects of these damaging variants on HBV predisposition. The novel rare variant +20988G > A, which was adjacent to the boundary of the splicing site between intron 4 and exon 5, occurred 10 times in the PSE + PS group and was absent in the controls. This mutant might affect the splicing efficiency of NTCP.

To summarize, we recruited 1075 population-based healthy control subjects who did not carry antibodies and antigens against HBV, although this was not easy, due to the popularity of the hepatitis B vaccine in China over the past 20 years. Using a special study design that included control, PS, PSE, and HCC groups, we failed to observe a dose effect of genetic variants on different traits but observed a trend towards the effects of genetic variants on different levels of the HBV infection status. However, the statistical power of the present study may be insufficient for variants with low frequencies.

## Conclusions

In this study, we found that the genetic variant rs4646287 located in intron 1 of *NTCP* was associated with the HBV infection status in a Han Chinese population. Additionally, the coding variants discovered in the sequenced subjects (especially the race-specific SNP rs2296651) may help elucidate the relationship between *NTCP* substrate transport and HBV binding and provide a candidate for functional analysis. More studies on functional analysis of these mutations are needed to determine whether our finding is useful for HBV drug target identification.

## Availability of supporting data

All primers used for Sanger sequencing [see Additional file 1] and variants reported in the manuscript [see Additional file 2] are provided in the Additional files.

## Additional files

Additional file 1: PCR and Sequencing primers in NTCP sequencing. The list of all primers used in PCR and sequencing in this manuscript, including the sequences, amplicons’ length and so on. (DOC 69 kb) Additional file 2: Information of 147 Variations observed in all 331 sequenced subjects. The list of all variations observed in all 331 sequenced subjects in this manuscript, including the locations, positions, observed minor allele frequencies and so on. (DOC 227 kb) Additional file 3: Distribution of Variants in *NTCP* in different groups. The MAFs of variants of each group (except for rs36115704 which has a much higher MAF of 49.6 %) show in different colors and symbols along their gene position: symbol “○” in black color, “*****” in cyan color and “●” in red color represent MAFs of control group, PS group and PSE group, respectively. The x axis indicates the physical position of the variants relative to the transcriptional initiation site (+1), and the y axis shows the values of within-group MAFs. The five colored boxes on the button of the plot represent the five exons of *NTCP* gene. The UTR regions show differently in blue with the coding regions show in orange. (TIFF 323 kb) Additional file 4: Clinical Characteristics and Outcome of 33 HBV-relative HCC patients according to the genotypes of rs4646287. Clinical Characteristics and Outcome of 33 HBV-relative HCC patients according to the genotypes of rs4646287 were decripted in this table. No significant differences of these clinical characteristics were observed between the two genotype groups of rs4646287. (DOC 40 kb) Additional file 5: NTCP mRNA expression levels in different genotypes of rs4646287. NTCP mRNA levels were lower in subjects with CT + TT genotypes than those with the CC genotype, although the difference was nearly 4.8 fold in tumor tissues but only 10 % in normal tissues. (DOC 50 kb)

## Abbreviations

PS: HBeAg-negativity HBsAg carriers
PSE: HBeAg-positivity HBsAg carriers
HCC: hepatocellular carcinoma
HBV: hepatitis B virus
SNP: single nucleotide polymorphisms
NTCP: sodium taurocholate cotransporting polypeptide
TBHBV: tent-making bat HBV
TZL: Taizhou longitudinal
MAF: minor allele frequency
