# Supplementary material for: A genetic variant of the NTCP gene is associated with HBV infection status in a Chinese population
Source: BMC Cancer. 2016 Mar 12;16:211. doi: 10.1186/s12885-016-2257-6 (PMC4788942; doi:10.1186/s12885-016-2257-6)
Supplement: Additional file 1: — PCR and Sequencing primers in NTCP sequencing. The list of all primers used in PCR and sequencing in this manuscript, including the sequences, amplicons’ length and so on. (DOC 69 kb) [file 12885_2016_2257_MOESM1_ESM.doc]

**Additional file 1: Table S1. PCR and Sequencing primers in NTCP sequencing**

| Amplican  Code | Forward primer sequence | Reverse primer sequence | Sequencing Primers | amplicon length | start | end |
| --- | --- | --- | --- | --- | --- | --- |
| N01 | GGTGGTGTCTTGACTCACTGGA | TTCGGGTATCAGTTTGGCAA | N01F | 1072 | 70240928 | 70241999 |
| N02 | TATGTAACCAAGTCTCCTAGCAGATG | ATCCCATTCCAAGTTTTGTGAC | NO2F | 852 | 70241782 | 70242633 |
| N03 | CTAACATGATGCATTCGCCCAGTT | CTTCATTGTTCACTCCTCTTGTTCTCT | N03F | 783 | 70242382 | 70243164 |
| N04 | GAGAAAGACTCAGGCAAGACTGGT | CACAGTGTATCTCAGAAGACCCCA | N04F | 856 | 70242896 | 70243751 |
| NO5 | TTGCATTCAGGGAATGAGGAG | CTACCCAAATCCTTCAACTGTCC | N05R | 813 | 70243413 | 70244225 |
| N06 | GCGTTCCTCTCAGATAAGCATCAG | CCAAGAAGCACCCAAGAAGCATTA | N06F&N06R | 1011 | 70243906 | 70244916 |
| N07 | TCCTTTACTGCGTAACTAGAGTTGAG | CTCTGAGTGTATGTGGGGTTTTC | N07R | 759 | 70244525 | 70245283 |
| N08 | CCTTGGGAGTCTTGAATTTCTCATAGC | GGTTTAACTGATCCTCCTGCCTCA | N08F | 752 | 70245050 | 70245801 |
| N09 | GAGCTGAGAATGTGCTACTCCCCT | CATTTTGCAGGAGTCCTGAATAGC | N09R | 725 | 70245823 | 70246547 |
| N10 | CACATTGATGGCAGAGAGAACTGT | AAACCGTATTTCCGGGCTTGATC | N10F | 763 | 70246015 | 70246777 |
| N11 | TGTTACGTGAGGCTATTCAGGACT | CTTAATGCCACTGAACTGTACACTCA | N11F | 768 | 70246512 | 70247279 |
| N12 | GCGTCCTCCAAAATGATAAACA | AGAGGCAGCTTTCCCACCAG | N12F | 758 | 70247030 | 70247787 |
| N13 | GCTAATGAACTTCTCAGTTTACCTTTGC | GCTATTGCTTCCCTCTCTGTAATCCT | N13F | 744 | 70247492 | 70248235 |
| N14 | ACCACATTTTGTACCACCTGACTTC | CCTTCGTCACCCTGTCCCATA | N14F | 735 | 70248030 | 70248764 |
| N15 | TTAGCCACATAGCCTTTGGATAAACC | CCACCACACCTTGCCGAGAA | N15F | 799 | 70248459 | 70249257 |
| N16 | TGGTTTAACCCTGGAGGTTTAGCC | AACTGGGAAACAGAATACCAAGTCATAG | N16F | 763 | 70248905 | 70249667 |
| N17 | GCTCTGCCAAGTAACTTTTCCG | TGATTTGGTTTCAACTGACCTCAC | N17R | 1196 | 70249131 | 70250326 |
| N18 | GCAGACAGGACTTGCCCTTCAT | ATGTGGTTATCTCAGTCGGTGGC | N18F | 758 | 70249902 | 70250659 |
| N19 | GGTGAGGTCAGTTGAAACCAA | CCCTCAGCAATTCGTTTCCA | N19F | 865 | 70250302 | 70251166 |
| N20 | TGCCTTTGACCAGAGGAACTCATAAG | CTTGTCCTGTCCCAGTGCCATG | N20F | 741 | 70250893 | 70251633 |
| N21 | GGGGAAATGGAAGGTGTTTGA | AACCTGGGGAGAAGCCTGAAT | N21R | 955 | 70251203 | 70252157 |
| N22 | GTTCTCAGCAGCACGCCTTG | ACGAAACCCCGTCTCCATTA | N22F | 785 | 70251741 | 70252525 |
| N23 | CTAAATGCCTCAAGCCCTTCC | ATGACCACCTGCTCCACCTTC | N23R | 858 | 70252161 | 70253018 |
| N24 | CTAATTTGTCAAGCCTCCCAGGTTCT | AAAGTCACTACAAGAAGCCATTCCCTA | N24F | 730 | 70252785 | 70253514 |
| N25 | CTTAGGGTGGTTCAACTGCTTCAATC | AGACAGGGTCCTGGGACTAACATAT | N25F | 754 | 70253267 | 70254020 |
| N26 | AGATAACCTGGTCAGATTTGCTTTG | CAGGGGTCTTACTTTGTTGCC | N26R | 779 | 70253800 | 70254578 |
| N27 | CCAGAGCGTAGCCATCAAGAACA | AAGAAACCATTGCCTAATCCAAAGTCC | N27R | 791 | 70254296 | 70255086 |
| N28 | CCAGAGCGTAGCCATCAAGAAC | CCACCACCAGTGTATGAGGATTC | N28R | 1538 | 70254296 | 70255833 |
| N29 | CGCCCGGCCAATATATGAAGAAC | GCCTTGCCAGAGGTGCCTATT | N29F | 1135 | 70255554 | 70256688 |
| N30 | AGGTTCACAGGCTTGGAATTATGC | TTTGCTGTGCTTACTAGGGCCTATA | N30F&N30R | 1176 | 70256337 | 70257512 |
| N31 | GAACGAAAGCTGGAGAATGATATTACG | TTACTATCTACAAGCCTGTCCAACATG | N31F& NEST1(CAATGATAGGATTGTCCACAGA)& NEST2(AGCACTTTGGGAGGTCAAG)& NEST3(GAGTGCAGTGGTGTGATCTTGG) | 1821 | 70257077 | 70258897 |
| N32 | CTCAACGTGGCTTGGAATCTTCTAT | TCCCTGTTTCTCTGTGCCTTCAT | N32F&N32R | 789 | 70258503 | 70259291 |
| N33 | GCTCCAAACTGTGAATAGACTGTTG | CAATCTCATTACCCTCCACCACTTG | N33F&N33R | 739 | 70259013 | 70259751 |
| N34 | CAGCAGTTCAGATACATGAGGTAGC | GCATTGACGGACACTCGAACG | N34R | 745 | 70259501 | 70260245 |
| N35 | CATCCGTGACTGTTGTGATCCTTG | CCTCCACTCACTCTGCCTCATAG | N35F | 729 | 70260015 | 70260743 |
| N36 | TGACAAGCCATACTCACTTCAACTG | TTCCTCTGTCTAATCCACATCCTCTC | N36F&N36R& NEST4(CTGTGTCTTACAATTCCTTCCAGTTCT) | 1378 | 70260540 | 70261917 |
| N37 | AGTCTGGCAGCACATGAAGGTA | ACGAGTTCAAGGCTACAGTGAGT | N38F | 867 | 70261349 | 70262215 |
| N38 | GAGAGGATGTGGATTAGACAGAGGAA | AACCTCAGTCTCAAGCCAGTCC | N38F&N38R | 756 | 70261892 | 70262647 |
| N39 | ACTCACTGTAGCCTTGAACTCGTA | AAGGTAGTGTGATTCGTTCCAAACA | N39R | 952 | 70262193 | 70263144 |
| N40 | CCTTATCAGGGTGGCTGAGATGG | GGCTGAAGAACATTGAGGCACTG | N40F | 743 | 70262880 | 70263622 |
| N41 | CCAGCATAAGCTCTTGGCAATAAATG | CCAGGAATCCAGCAGAGGTCAG | N41R | 770 | 70263353 | 70264122 |
| N42 | TCCTGTGAGGCAGTGGAAGAC | GCAGCCACCACCAGTATTCCT | N42R | 732 | 70263875 | 70264606 |
