# Supplementary material for: A genetic variant of the NTCP gene is associated with HBV infection status in a Chinese population
Source: BMC Cancer. 2016 Mar 12;16:211. doi: 10.1186/s12885-016-2257-6 (PMC4788942; doi:10.1186/s12885-016-2257-6)
Supplement: Additional file 2: — Information of 147 Variations observed in all 331 sequenced subjects. The list of all variations observed in all 331 sequenced subjects in this manuscript, including the locations, positions, observed minor allele frequencies and so on. (DOC 227 kb) [file 12885_2016_2257_MOESM2_ESM.doc]

**Additional file 2 Table S2** Information of 147 Variations observed in all 331 sequenced subjects

| **rs#（Reported）**  **/site#（Novel）** | **Position  on CHR14(+)** | **Position refer**  **to transcription**  **site（+1）** | **Location** | **AA_change** | **MAF (All 331)** |
| --- | --- | --- | --- | --- | --- |
| **SITE1** | 70241049:A/G | +23062 A/G | 3Downs |  | 0.16% |
| **SITE2** | 70241137:A/T | +22974 A/T | 3Downs |  | 0.33% |
| **rs11628646** | 70241380:A/C | +22731 A/C | 3Downs |  | 0.49% |
| **SITE3** | 70241398:T/C | +22713 T/C | 3Downs |  | 0.17% |
| **SITE4** | 70241454:-/C | +22657 -/C | 3Downs |  | 0.65% |
| **SITE5** | 70241488:G/C | +22623 G/C | 3Downs |  | 0.16% |
| **SITE6** | 70241540:T/A | +22571 T/A | 3Downs |  | 0.33% |
| **rs11628755** | 70241600:C/T | +22511 C/T | 3Downs |  | 0.33% |
| **SITE7** | 70241642:T/C | +22469 T/C | 3Downs |  | 0.16% |
| **rs61982104** | 70241661:A/G | +22450 A/G | 3Downs |  | 0.32% |
| **rs11628839** | 70241663:G/A | +22448 G/A | 3Downs |  | 0.32% |
| **rs141438605** | 70241843:G/T | +22268 G/T | 3Downs |  | 3.70% |
| **rs28595072** | 70241961:C/T | +22150 C/T | 3Downs |  | 1.07% |
| **rs4646297** | 70241993:C/T | +22118 C/T | 3Downs |  | 16.62% |
| **rs143440974** | 70242003:A/G | +22108 A/G | 3Downs |  | 7.01% |
| **SITE8** | 70242012:T/- | +22099 T/- | 3Downs |  | 0.15% |
| **SITE9** | 70242363:C/T | +21748 C/T | 3'UTR |  | 0.15% |
| **SITE10** | 70243052:C/T | +21059 C/T | exon5 | 333A>T | 0.15% |
| **SITE11** | 70243082:C/G | +21029 C/G | exon5 | 323A>P | 0.15% |
| **SITE12** | 70243123:G/A | +20988 G/A | intron4 |  | 0.47% |
| **SITE13** | 70243137:G/C | +20974 G/C | intron4 |  | 0.15% |
| **rs4646296** | 70243505:C/G | +20606 C/G | intron4 |  | 8.84% |
| **SITE14** | 70243532:G/A | +20579 G/A | intron4 |  | 0.15% |
| **SITE15** | 70243792:A/G | +20319 A/G | intron4 |  | 0.15% |
| **rs9323529** | 70243813:T/G | +20298 T/G | intron4 |  | 0.15% |
| **SITE16** | 70244737:C/T | +19374 C/T | intron4 |  | 0.18% |
| **rs182004992** | 70244816:A/G | +19295 A/G | intron4 |  | 0.68% |
| **rs2296651** | 70245193:G/A | +18918 G/A | exon4 | 267S>F | 1.39% |
| **SITE17** | 70245226:A/G | +18885 A/G | exon4 | 256M>T | 0.20% |
| **rs56063215** | 70245407:A/G | +18704 A/G | intron3 |  | 0.93% |
| **SITE18** | 70245538:C/G | +18573 C/G | intron3 |  | 0.19% |
| **rs180876096** | 70245550:G/A | +18561 G/A | intron3 |  | 0.37% |
| **SITE19** | 70245831:A/C | +18280 A/C | intron3 |  | 0.16% |
| **SITE20** | 70245980:A/G | +18131 A/G | exon3 | 222L>S | 0.16% |
| **rs202213974** | 70246047:C/T | +18064 C/T | exon3 | 200V>M | 0.16% |
| **SITE21** | 70246177:A/G | +17934 A/G | intron2 |  | 0.15% |
| **rs139537133** | 70246217:TCT/- | +17894 TCT/- | intron2 |  | 0.92% |
| **rs116084010** | 70246345:G/A | +17766 G/A | intron2 |  | 0.15% |
| **rs55724465** | 70246412:G/A | +17699 G/A | intron2 |  | 15.64% |
| **rs11626135** | 70246449:C/G | +17662 C/G | intron2 |  | 0.77% |
| **SITE22** | 70246544:A/G | +17567 A/G | intron2 |  | 0.16% |
| **rs113492713** | 70246554:-/GAGGGT | +17557  -/GAGGGT | intron2 |  | 15.10% |
| **rs28437822** | 70246718:A/G | +17393 A/G | intron2 |  | 15.56% |
| **SITE23** | 70246770:-/A | +17341 -/A | intron2 |  | 0.44% |
| **SITE24** | 70246956:A/G | +17155 A/G | intron2 |  | 0.22% |
| **SITE25** | 70247033:C/T | +17078 C/T | intron2 |  | 0.23% |
| **rs117750794** | 70247081:A/G | +17030 A/G | intron2 |  | 3.79% |
| **SITE26** | 70247100:AC/- | +17011 AC/- | intron2 |  | 0.16% |
| **rs111409076** | 70247103:-/AACA | +17008 -/AACA | intron2 |  | 2.17% |
| **SITE27** | 70247170:TCAC/- | +16941 TCAC/- | intron2 |  | 0.16% |
| **SITE28** | 70247211:AAA/- | +16900 AAA/- | intron2 |  | 0.62% |
| **rs78844923** | 70247219:AT/- | +16892 AT/- | intron2 |  | 0.16% |
| **SITE29** | 70247267:C/G | +16844 C/G | intron2 |  | 0.16% |
| **SITE30** | 70247612:G/A | +16499 G/A | intron2 |  | 0.19% |
| **rs3784152** | 70247768:T/A | +16343 T/A | intron2 |  | 15.91% |
| **rs3784153** | 70247782:C/G | +16329 C/G | intron2 |  | 16.08% |
| **rs75781181** | 70247842:G/A | +16269 G/A | intron2 |  | 0.35% |
| **SITE31** | 70248136:T/C | +15975 T/C | intron2 |  | 0.16% |
| **rs17556915** | 70248358:G/A | +15753 G/A | intron2 |  | 0.16% |
| **SITE32** | 70248630:A/G | +15481 A/G | intron2 |  | 0.16% |
| **SITE33** | 70248779:G/C | +15332 G/C | intron2 |  | 0.17% |
| **SITE34** | 70248787:A/G | +15324 A/G | intron2 |  | 0.17% |
| **rs10459536** | 70249246:A/C | +14865 A/C | intron2 |  | 16.77% |
| **SITE35** | 70249263:G/A | +14848 G/A | intron2 |  | 0.16% |
| **rs10130174** | 70249309:G/C | +14802 G/C | intron2 |  | 0.93% |
| **SITE36** | 70249398:G/A | +14713 G/A | intron2 |  | 0.16% |
| **rs192088710** | 70249948:G/C | +14163 G/C | intron2 |  | 0.77% |
| **SITE37** | 70250023:A/G | +14088 A/G | intron2 |  | 0.15% |
| **rs11624523** | 70250500:A/G | +13611 A/G | intron2 |  | 16.52% |
| **rs147575974** | 70250725:C/T | +13386 C/T | intron2 |  | 0.36% |
| **rs17107282** | 70250767:C/T | +13344 C/T | intron2 |  | 0.89% |
| **SITE38** | 70251056:C/T | +13055 C/T | intron2 |  | 0.19% |
| **SITE39** | 70251160:T/C | +12951 T/C | intron2 |  | 0.36% |
| **rs3837667** | 70251238:AGAG/- | +12873 AGAG/- | intron2 |  | 1.61% |
| **SITE40** | 70251283:G/T | +12828 G/T | intron2 |  | 0.17% |
| **SITE41** | 70251453:G/A | +12658 G/A | intron2 |  | 0.17% |
| **rs3837668** | 70251494:T/- | +12617 T/- | intron2 |  | 17.16% |
| **rs59017109** | 70251593:C/T | +12518 C/T | intron2 |  | 3.05% |
| **SITE42** | 70251775:G/A | +12336 G/A | intron2 |  | 0.15% |
| **rs184422099** | 70251925:A/G | +12186 A/G | intron2 |  | 0.15% |
| **SITE43** | 70252008:T/C | +12103 T/C | intron2 |  | 0.15% |
| **rs72725740** | 70252100:A/G | +12011 A/G | intron2 |  | 15.34% |
| **SITE44** | 70252605:C/T | +11506 C/T | intron2 |  | 0.15% |
| **SITE45** | 70252631:G/A | +11480 G/A | intron2 |  | 0.15% |
| **SITE46** | 70252990:G/C | +11121 G/C | Exon2 | 131L>V | 0.17% |
| **rs76091563** | 70253049:G/T | +11062 G/T | intron1 |  | 0.17% |
| **SITE47** | 70253058:A/C | +11053 A/C | intron1 |  | 0.17% |
| **rs190547040** | 70253183:C/T | +10928 C/T | intron1 |  | 1.20% |
| **SITE48** | 70253354:C/T | +10757 C/T | intron1 |  | 0.16% |
| **SITE49** | 70253556:T/A | +10555 T/A | intron1 |  | 0.16% |
| **SITE50** | 70253806:C/G | +10305 C/G | intron1 |  | 0.16% |
| **rs35161261** | 70253914:CACT/- | +10197 CACT/- | intron1 |  | 0.15% |
| **rs60915717** | 70253916:C/T | +10195 C/T | intron1 |  | 0.15% |
| **SITE51** | 70253933:A/G | +10178 A/G | intron1 |  | 0.15% |
| **rs2332167** | 70254327:G/A | +9784 G/A | intron1 |  | 16.05% |
| **SITE52** | 70254387:C/T | +9724 C/T | intron1 |  | 0.16% |
| **SITE53** | 70254609:C/A | +9502 C/A | intron1 |  | 0.32% |
| **SITE54** | 70254692:G/A | +9419 G/A | intron1 |  | 0.32% |
| **SITE55** | 70255301:G/A | +8810 G/A | intron1 |  | 0.17% |
| **rs183757760** | 70255332:G/T | +8779 G/T | intron1 |  | 0.16% |
| **SITE56** | 70255423:G/A | +8688 G/A | intron1 |  | 4.11% |
| **rs11621916** | 70255617:A/G | +8494 A/G | intron1 |  | 15.70% |
| **SITE57** | 70255827:G/T | +8284 G/T | intron1 |  | 0.16% |
| **SITE58** | 70255858:T/C | +8253 T/C | intron1 |  | 0.16% |
| **SITE59** | 70256002:G/A | +8109 G/A | intron1 |  | 0.17% |
| **rs79045137** | 70256085:T/C | +8026 T/C | intron1 |  | 0.17% |
| **rs58582364** | 70256407:G/T | +7704 G/T | intron1 |  | 15.14% |
| **rs8020042** | 70257369:A/G | +6742 A/G | intron1 |  | 0.84% |
| **SITE60** | 70257393:C/T | +6718 C/T | intron1 |  | 0.17% |
| **SITE61** | 70257409:A/G | +6702 A/G | intron1 |  | 0.17% |
| **SITE62** | 70257427:TAAC/- | +6684 TAAC/- | intron1 |  | 0.17% |
| **rs11624532** | 70257661:C/T | +6450 C/T | intron1 |  | 14.80% |
| **SITE63** | 70257705:A/- | +6406 A/- | intron1 |  | 0.65% |
| **rs72725745** | 70258001:G/A | +6110 G/A | intron1 |  | 14.88% |
| **rs182117739** | 70258239:G/A | +5872 G/A | intron1 |  | 3.64% |
| **rs10142027** | 70258263:A/C | +5848 A/C | intron1 |  | 15.27% |
| **rs10142031** | 70258270:A/G | +5841 A/G | intron1 |  | 0.81% |
| **rs192746790** | 70258453:C/G | +5658 C/G | intron1 |  | 0.32% |
| **SITE64** | 70258911:GAAA/- | +5200 GAAA/- | intron1 |  | 0.15% |
| **SITE65** | 70259012:A/T | +5099 A/T | intron1 |  | 0.31% |
| **rs8011311** | 70259063:G/C | +5048 G/C | intron1 |  | 16.41% |
| **SITE66** | 70259441:G/A | +4670 G/A | intron1 |  | 0.16% |
| **SITE67** | 70259540:G/T | +4571 G/T | intron1 |  | 0.16% |
| **SITE68** | 70259800:G/A | +4311 G/A | intron1 |  | 0.16% |
| **SITE69** | 70260086:A/G | +4025 A/G | intron1 |  | 0.15% |
| **rs3784154** | 70260226:G/A | +3885 G/A | intron1 |  | 1.59% |
| **rs202150129** | 70260412:C/- | +3699 C/- | intron1 |  | 0.63% |
| **SITE70** | 70260477:G/T | +3634 G/T | intron1 |  | 0.16% |
| **SITE71** | 70260617:A/C | +3494 A/C | intron1 |  | 0.16% |
| **rs189073029** | 70260829:T/C | +3282 T/C | intron1 |  | 1.33% |
| **rs943276** | 70260930:G/A | +3181 G/A | intron1 |  | 0.34% |
| **rs10134553** | 70261084:G/C | +3027 G/C | intron1 |  | 0.16% |
| **rs943277** | 70261325:G/A | +2786 G/A | intron1 |  | 0.47% |
| **rs145260192** | 70261514:-/A | +2597 -/A | intron1 |  | 0.91% |
| **SITE72** | 70261531:G/A | +2580 G/A | intron1 |  | 0.15% |
| **rs11622925** | 70261665:C/T | +2446 C/T | intron1 |  | 15.30% |
| **SITE73** | 70261753:A/G | +2358 A/G | intron1 |  | 0.15% |
| **rs36115704** | 70262419:C/T | +1692 C/T | intron1 |  | 46.97% |
| **rs144058121** | 70262803:C/T | +1308 C/T | intron1 |  | 0.15% |
| **rs4646287** | 70262815:C/T | +1296 C/T | intron1 |  | 10.03% |
| **rs4646286** | 70262910:C/G | +1201 C/G | intron1 |  | 16.87% |
| **rs117354704** | 70263489:G/T | +622 G/T | intron1 |  | 0.31% |
| **rs148467625** | 70263610:A/G | +501 A/G | exon1 | 88I>T | 0.31% |
| **rs4646285** | 70263648:C/T | +463 C/T | exon1 | 75T>T | 14.97% |
| **SITE74** | 70263710:A/G | +401 A/G | exon1 | 55L>L | 0.15% |
| **TMP_ESP_**  **14_70263874**  **（no rs#）** | 70263874:C/T | +237 C/T | 5'UTR |  | 7.77% |
| **SITE75** | 70264549:G/A | -439 G/A | 5ups |  | 0.63% |

Note: Variants’ names beginning with SITE means the variants that didn’t appear in dbSNP.
