# Supplementary figures and images for: A genetic variant of the NTCP gene is associated with HBV infection status in a Chinese population
Source: BMC Cancer. 2016 Mar 12;16:211. doi: 10.1186/s12885-016-2257-6 (PMC4788942; doi:10.1186/s12885-016-2257-6)

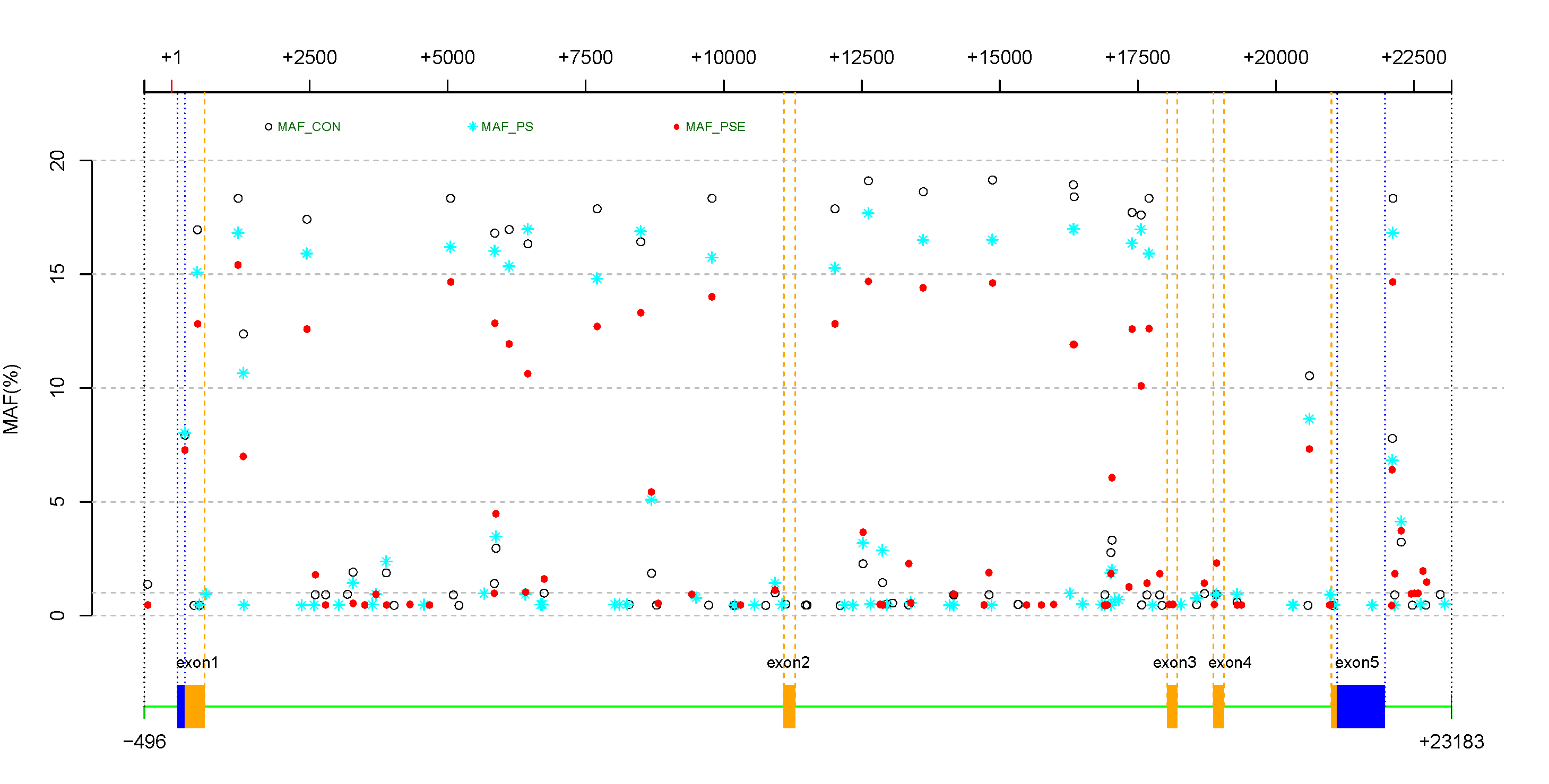

Supplement: Additional file 3: — Distribution of Variants in NTCP in different groups. The MAFs of variants of each group (except for rs36115704 which has a much higher MAF of 49.6 %) show in different colors and symbols along their gene position: symbol “○” in black color, “*” in cyan color and “●” in red color represent MAFs of control group, PS group and PSE group, respectively. The x axis indicates the physical position of the variants relative to the transcriptional initiation site (+1), and the y axis shows the values of within-group MAFs. The five colored boxes on the button of the plot represent the five exons of NTCP gene. The UTR regions show differently in blue with the coding regions show in orange. (TIFF 323 kb) [file 12885_2016_2257_MOESM3_ESM.tiff]
