# Supplementary material for: A genetic variant of the NTCP gene is associated with HBV infection status in a Chinese population
Source: BMC Cancer. 2016 Mar 12;16:211. doi: 10.1186/s12885-016-2257-6 (PMC4788942; doi:10.1186/s12885-016-2257-6)
Supplement: Additional file 4: — Clinical Characteristics and Outcome of 33 HBV-relative HCC patients according to the genotypes of rs4646287. Clinical Characteristics and Outcome of 33 HBV-relative HCC patients according to the genotypes of rs4646287 were decripted in this table. No significant differences of these clinical characteristics were observed between the two genotype groups of rs4646287. (DOC 40 kb) [file 12885_2016_2257_MOESM4_ESM.doc]

**Additional file 4: Table S3** Clinical Characteristics and Outcome of 33 HBV-relative HCC patients according to the genotypes of rs4646287

|  | Rs4646287 | |  |
| --- | --- | --- | --- |
|  | CC | CT+TT | P value |
| All Cases | 16 | 17 |  |
| Gender |  |  | 0.164 |
| Male | 12 | 16 |  |
| Female | 4 | 1 |  |
| AFP |  |  | 0.282 |
| <400 | 12 | 9 |  |
| >400 | 4 | 8 |  |
| Size |  |  | 0.688 |
| <3cm | 3 | 5 |  |
| >3cm | 13 | 12 |  |
| BCLC |  |  | 0.652 |
| A | 5 | 2 |  |
| B or C | 10 | 10 |  |
| TNM |  |  | 1 |
| T1/T2 | 13 | 14 |  |
| T3/T4 | 2 | 1 |  |
| Liver cirrhosis | |  | 0.589 |
| Yes | 13 | 16 |  |
| No | 2 | 1 |  |
