# Supplementary material for: A genetic variant of the NTCP gene is associated with HBV infection status in a Chinese population
Source: BMC Cancer. 2016 Mar 12;16:211. doi: 10.1186/s12885-016-2257-6 (PMC4788942; doi:10.1186/s12885-016-2257-6)
Supplement: Additional file 5: — NTCP mRNA expression levels in different genotypes of rs4646287. NTCP mRNA levels were lower in subjects with CT + TT genotypes than those with the CC genotype, although the difference was nearly 4.8 fold in tumor tissues but only 10 % in normal tissues. (DOC 50 kb) [file 12885_2016_2257_MOESM5_ESM.doc]

**Additional file 5: Table S4** NTCP mRNA expression levels in different genotypes of rs4646287

| Relative Expression | Tissues | rs4646287 | N | Mean±SD | p |
| --- | --- | --- | --- | --- | --- |
| NTCP/Reference (Mean of  ACTB and TBP) | Normal | CC | 16 | 3.07±1.04 | 0.318 |
|  |  | CT+TT | 17 | 2.69±1.10 |  |
|  | Tumor | CC | 16 | 1.18±1.51 | 0.010 |
|  |  | CT+TT | 17 | 0.18±0.16 |  |
|  | Tumor/Normal | CC | 16 | 0.36±0.40 | 0.009 |
|  |  | CT+TT | 17 | 0.08±0.09 |  |
| NTCP/ACTB | Normal | CC | 16 | 0.44±0.21 | 0.588 |
|  |  | CT+TT | 17 | 0.40±0.18 |  |
|  | Tumor | CC | 16 | 0.14±0.15 | 0.005 |
|  |  | CT+TT | 17 | 0.03±0.03 |  |
|  | Tumor/Normal | CC | 16 | 0.35±0.37 | 0.008 |
|  |  | CT+TT | 17 | 0.08±0.10 |  |
| NTCP/TBP | Normal | CC | 16 | 22.81±8.69 | 0.172 |
|  |  | CT+TT | 17 | 18.63±8.50 |  |
|  | Tumor | CC | 16 | 10.50±15.19 | 0.022 |
|  |  | CT+TT | 17 | 1.54±1.94 |  |
|  | Tumor/Normal | CC | 16 | 0.41±0.50 | 0.023 |
|  |  | CT+TT | 17 | 0.11±0.15 |  |
